# Cases of a Fœtus Found in the Abdomen of a Young Man, at Sherborne, in Dorsetshire

**Published:** 1816-01

**Authors:** 


*Case of a Fœtus found in the Abdomen of a young Man, at Sherborne, in Dorsetshire.*

By NATHANIEL HIGHMORE, Surgeon, and Member of the Royal College of Surgeons, London. 4to. pp. 30, with explanatory Engravings. 1815.

WE entered upon the examination of this fair and elegant quarto in a somewhat unpropitious mood. If ever Mr. Highmore have been doomed to listen, for two irksome hours, to the moving tale of symptoms of some nervous and fantastic maiden lady, devoured by indigestion and ennui, while an interesting case of aortic aneurism awaited his observation in the next room, he will be able to comprehend, more distinctly than language can describe, the feeling of fretful reluctance with which we relinquished the instructive lessons of Orfila for the popular description of the wonderful fœtus.

The thing, however, is curious; and our duty, as public Journalists, requires that an account of the monster, observed by Mr. Highmore, be duly recorded. But we could have wished that his narrative had come before us in less pompous and costly array, in the humble garb and character suited to its intrinsic merits—to the value which will be stamped upon it, not by dolts and visionaries, but by enlightened and philosophic men.

Yet it is gratifying to find, that Mr. Highmore, extravagantly as the importance of his subject, and the responsibility of the situation to which he is "involuntarily" called, have been viewed beneath the microscope of an ardent imagination, seized not on the unfortunate fœtus as a mean whereby his own extraordinary talents might be blazoned to the world, as the vehicle of disgusting egotism. Rarely, indeed, have we perused a more modest and unaffected narrative. There exist throughout the whole, a candour, an earnestness, a simplicity, exceedingly honourable to his character and feelings, and worthy of a deeper and more instructive topic.

Thrice, within the last ten years, has an organized mass, possessing more or less perfectly the characters of the human fœtus, been found embowelled in the male subject.* By presenting a connected view of these several phænomena in the respective order of their occurrence, we shall, perchance, render an acceptable service to those of our readers who possess not opportunity or inclination to consult the works in which they are originally consigned. Thus too will they be enabled at once to appreciate the peculiarities of structure and circumstance by which these monstrous productions are distinguished; and seize, without farther labour or research, the points of resemblance which serve to connect them.

*Case by Mons. Dupuytren.*†—Amédée Bissieu, a boy in whose sexual organs no ambiguous feature prevailed, was affected *from infancy* with pain and tumor of the left side. At the age of thirteen, he suffered an attack of fever: the tumor, from that period, became large and painful; his fæces offensive and purulent. After a lapse of three months, symptoms of pulmonary phthisis were developed. A mass of human hair was voided by the rectum; and six weeks afterwards, about the age of fourteen years, the boy died.—*On dissection*, a sac was found situated in the transverse mesocolon; adhering to the arch of the colon; and communicating, by an orifice of recent formation, with the cavity of that intestine. The cyst contained

* Several analogous cases are on record. We select the two immediately preceding that of Mr. Highmore, from their recent occurrence, and the high character of the gentlemen by whom they are attested. They, moreover, amply suffice for the purposes of comparison; and characters of organization so numerous and strongly marked are displayed in both these monstrous productions, as to leave on the unprejudiced mind no doubt of their real nature.

† Bulletin de l'Ecole de Médecine de Paris, et de la Societé établie dans son Sein. Première Année, No. 1, An. XIII.

balls of hair and distinct traces of a human fœtus. The short umbilical chord was inserted into the mesocolon; it consisted of an artery and vein ramifying both at the mesocolic and fœtal extremities. An osseous system, composed of cranium, vertebral column, pelvis, and rudiments of limbs; degenerated muscles; brain, spinal marrow, and nerves, were clearly distinguished. The digestive and respiratory organs, with those of urine and generation, were deficient.

*Mr. Young's Case.**—John Hare, born May 18th, 1807, healthy and well formed, was subject, *nearly from his birth*, to bilious vomitings. A tumor shortly afterwards appeared, somewhat to the left of the *scrobiculus cordis.* When first seen in September by Mr. Young, he preserved his healthy appearance: vomitings recurring at intervals; stools green; sleep disturbed; pain considerable. The tumor, occupying the epigastric and umbilical regions with an inclination to the left side, was moveable, tense, and distinctly fluctuated at its most prominent part. From this time the child grew thinner; the pain and tumor increased. For a week preceding the 23d of December, his sufferings were constant and severe. On that day, a remarkable change took place in the form and resistance of the tumor. The abdomen became soft; the child easy; large quantities of urine were voided. The vomiting ceased. His strength, appetite, and appearance improved for a while: but the cyst, which had evidently ruptured and discharged its contents into the peritonæum, soon filled again. On January 7th, he was greatly emaciated. The abdomen was again large; and a hard tumor could be felt floating in a cyst not firmly distended by fluid. The abdomen continued to increase; vomiting returned. Rest and appetite were again lost. Emaciation became extreme. He died on the 25th of February.—*Appearances on dissection.* A large, spherical, somewhat transparent tumor, occupying the abdomen, and situated between the laminæ of the transverse mesocolon; which, with the two omenta, the stomach, duodenum, pancreas, splenic branch of the *vena portarum*, and colon, passed over it anteriorly: in contact with it posteriorly, were the aorta, cœliac and superior mesenteric arteries, left crus of the diaphragm, and termination of the duodenum. Its inferior part rested on the mesentery. The small intestines were pushed down into

* Medico-Chirurgical Transactions, vol. i.

the pelvis. The cyst contained seventy-eight ounces of limpid fluid, and a human fœtus.—External characters. A dark, red, fleshy mass in place of head, resembling *pia mater,* plentifully supplied with blood-vessels, furnished with two locks of fine long hair, and connected by a slip of *dura mater* to the containing cyst; spine much curved. Two superior extremities: right, with three incomplete fingers; left, more perfectly formed, with two fingers. Two lower extremities: right, distinctly formed, foot furnished with eight toes; left, more defective, foot with five toes much mis-shapen. Breech well formed; clutch distinctly marked; no anus: penis, with its prepuce, glans, and an orifice not passing inwards more than a line: scrotum divided below. A fleshy cone, in reality an exomphalos, connecting the fœtus at the umbilicus to its cyst: a dark, red, denuded surface, broad above, pointed below, occupying the middle of the posterior region of the body from the shoulders to the sacrum: two eminences growing on the thorax.* Internal organization: Osseous system: a portion of the base of the skull; vertebral column without processes or canal; few ribs, very short; pelvis regularly formed; and extremities. Many of the bones and their apophyses cartilaginous: several of the joints well formed, with intervening cartilage, ligaments, and synovia. Very little muscular fibre; no diaphragm. No cerebral substance, spinal marrow, nerves of sense or voluntary motion; but a distinct nervous plexus within the umbilicus about the commencement of the intestines, and supplying them. Arterial system without a heart, consisting of two trunks; one conveying the blood from the cyst to the fœtus; the other returning it: no communication between the trunks, or them and the vessels of the containing child, detected. A slight trace of lungs: no liver, spleen, kidnies, bladder, or internal organs of generation: intestinal canal curiously constructed, filling the pelvis, passing into the exomphalos, and terminating in an anus at the right side, near the umbilicus. The cyst composed of two coats, unequally thick, performing the office of a placenta; thickest at the insertion of the exomphalos, and supplied by a large branch from the *arteria colica sinistra* of the containing child: vestiges of laceration seen on the internal membrane; and a hole on the corresponding part of the external.

---

* It is remarkable that an eminence, somewhat similar, was observed on the thorax of Mr. Highmore's fœtus.

*Mr. Highmore's Case.*—T. Lane, born Dec. 21st, 1798, and at first healthy, was seized, about his seventh year, with pains in the bowels, which were relieved by vermifuge medicines; but no worms were voided. At this time, the body was affected with considerable swelling, which again subsided as he grew better; but a visible enlargement remained. After this, he continued restless, occasionally delirious at night; and invariably, when in pain, referred it to his belly. During the six ensuing years, he went on favourably; and was attacked, in March 1811, with violent pain in the bowels: the swelling, which had existed from the period of the former attack, now increased, and again subsided in a week. Continuing afterwards unusually healthy for one year, he was seized, in April 1812, with great pain in the back and loins; but resumed, after a few days, his employment as carter's boy. In January 1814, from exposure to wet and cold, he was attacked with shiverings and pain in the legs, back, and loins. The abdominal enlargement was now gradually declining. Anthelmintics were again administered in February without effect. In March, diarrhœa came on, recurring every afternoon, and accompanied by violent pains in the abdomen and disposition to syncope. The lad himself reported that "blood and fleshy matter" were now continually evacuated by stool.

One day, about this time, he exclaimed in affright, "Mother, do come to me, I have something *alive* in my body;" and directly fainted. The mother declares, that she not only felt, but saw, a motion in the tumor resembling the motion of a child in gestation.

On May 9th, 1814, Mr. Highmore was first consulted. The situation of the poor lad was then deplorable. The abdomen presented the appearance of dropsy, and *fluctuation was perceptible*. There was, besides, an evident projection in the situation of the spleen; sore on pressure, and pulsating violently.* A motion, independent of this, was also felt, as though the part were "affected by spasmodic action." The lower extremities were œdematous; visage pale; pulse weak and irregular, 120 in the minute; bowels regular; urine pale and scanty. June 9th, considerable quantity of thin clotted blood was vomitted; the lad faint, and in great pain. Towards evening, the body

* This pulsation was probably communicated to the tumor by the subjacent aorta. Mr. Highmore should have mentioned whether or not it were synchronous with the stroke of the heart.

was covered with profuse perspiration; pulse very rapid: long pieces of coagulated blood, firm, elastic, and bearing the impression of the intestinal convolutions, were voided per anum. He expired, during the night, in extraordinary agony.

The spleen was regarded by Mr. Highmore as the seat of the morbid enlargement. The remedies employed were mercurial purgatives, nitrate of potash, squill, and opium, internally; mercurial frictions, externally. They were productive, for a time, of signal relief.

We deem it best to present, in Mr. Highmore's own words, his statement of the appearances displayed on

"*Examination of the Body.*

"On dividing the parietes of the abdomen, and exposing its viscera, a large tumor, of an irregular but somewhat oval form, presented itself. It occupied portions of the epigastric, umbilical, and left hypochondriac regions; and was uncovered by the omentum, which was found in a ruffled state, lying above the tumor."

"In tracing the course of the intestines upwards, and examining more particularly the tumor, I discovered that the jejunum was continued into the anterior and sinister-lateral-inferior part of the sac, inclosing the substance, of which, by its apparent expansion, it evidently formed a part. I then traced the course of the duodenum from the pyloric orifice of the stomach, and observed, that its whole curve was firmly attached to and connected with the sac, and that this intestine also terminating in it, formed the anterior and dexter-lateral portion."

"The spleen, which had previously been suspected to be the viscus diseased, was lying behind the tumor; appeared compressed by it, and was inflamed at its lower edge. The liver was perfectly sound and healthy, as were also the kidnies, ureters, and urinary bladder. There was no bile in the gall-bladder, nor scarcely any fæces in the intestines; the latter containing little more than a quantity of coagulated blood, with two or three slight appearances of inflammation, in the course of the jejunum and ileum."

"The young man, during the earlier part of my attendance on him, having laboured under frequent intermissions of pulse, I examined also the state of the thoracic viscera. I found the pulmonary organs, though not actually diseased, were of a bad colour, but perfectly capable of performing their functions. The pericardium was considerably distended, and contained about seven ounces of serous fluid; the heart was of the natural size, and quite free from disease. It was now too late in the evening to examine the tumor with the minuteness I had intended; I determined, therefore, with the mother's consent, to remove it entirely to my own house, there to inspect it, in conjunction with Mr. Gray, on the following morning."

" As this tumour appeared to be the only diseased part, and was evidently contained within the intestine, with a free communication between it, the duodenum, and the jejunum, I proceeded to remove it from the body; and in order to prevent any alimentary, or other matter from escaping, I secured the jejunum, below the tumor, with two ligatures, and divided it between them; but as the stomach was so closely connected with the tumor above, I considered that one ligature would there be sufficient, which I applied round the pyloric orifice, and divided the neck of the stomach. The only attachments that now remained were, to the mesentery, which had confined it close to the spine, and to the pancreas. I divided the mesentery; but as the pancreas so strongly adhered to it, immediately over the vertebræ, a portion of it was removed with the tumor."

" Having sewed up the body, I conveyed the tumor to my house, and found its weight to be four pounds and a half; and, whilst in the act of placing it in a vessel, where it was to remain during the night, my fore-finger accidentally slipped round the curve of the arm, at the elbow-joint, which first gave me the idea of its partaking of an animal form. At this circumstance I felt greatly astonished; and the more so, when, on my endeavouring to trace, through the sac, the extremity of the limb, I could count five digitals!"

" I instantly dispatched Mr. Eastment (who was also present during the whole examination,) for Mr. Gray—these gentlemen returned in a few minutes; when, having placed the tumor on the table, I opened the sac, at the contents of which we were amazed! We found that the substance assumed, in many respects, a completely human form; but in others, it was cramped and mis-shapen, as will appear by a reference to the plates and the accompanying description." Page 19.

Mr. Highmore and his medical friends, having by cautious examination of the lad's body, perfectly satisfied themselves as to his sex, proceeded on the following morning to investigate more minutely the contents of the cyst.

" *Description of the Fœtus, and of the sac or cyst in which it was contained.*

" On opening the sac by an oblique incision, through the anterior and thinnest part, an imperfect human fœtus was discovered. It lay on its left side, with the right arm, which was perfect, bent down upon the right hip; the left, which was very imperfectly formed, was resting against the leg, the thigh of which was bent up towards the abdomen; and the leg was bent at an acute angle upon the thigh."

" The fœtus was connected with the sac by a short, thick funis, which arose from its abdomen, rather towards the left side, passed forwards between the left arm and leg, and entered the sac at the posterior and upper part. It had no head; but at the basis of a denuded first vertebra some slips of skin arose, which followed nearly the course of the funis, with some medullary substance, around which was entangled a considerable quantity of matted hair, part of which measured twelve inches in length. There was also adhering to this skin a thin piece of bone, appearing to be a portion of cranium. The spine was much curved. There were two superior extremities, the right of which was perfectly formed, but had been fractured just above the carpal bones: the upper end of the radius was also dislocated, and protruded through the integuments. The left upper extremity was less perfectly formed; it was short, ill-shaped, and had only three fingers, with very long nails. The scapula of this arm rested on the side, almost as low down as the hip."

"There was only one inferior extremity; the thigh was bent forwards upon the body of the child, and the leg bent, sharp-angle, upon the thigh: the skin being common to each almost half their length, forming a kind of web, uniting the two together. The knee was dislocated; the skin over it had been absorbed, and the joint was exposed. The ankle was also dislocated, and turned inwards; the common integuments had been absorbed, and the bones were exposed and perishing. It had six imperfectly formed toes."

"The left inferior extremity was wanting; but there was a considerable surface divested of skin, at the spot where it should have been. This surface was regular and smooth, without any projecting bone, excepting at the inferior part, where a portion of ischium protruded."

"There was a quantity of sebaceous matter between the extremities and the body of the fœtus: on the upper part of the thorax was a long fleshy excrescence, somewhat like the papilla of an elderly woman; and the appearance of the genitals favoured the idea of the fœtus being a female."

"The sac, which contained the fœtus, was made up of two distinct portions: the larger portion, which was thick, spongy, and highly vascular, involved the greater part of the fœtus. A small portion of the buttocks, the bent carpus and the right arm, together with the foot and ankle, were covered by, and laying in contact with the inner surface of the intestine of the boy, which formed the second portion of the sac."

"The opening into the intestine seems to have been partly into the duodenum, and partly into the jejunum. The duodenum entered the cyst, the *posterior part having been ruptured, while the anterior was continued into the jejunum*, and formed the small portion of the described cyst. The jejunum emerged out of the sac, with a somewhat similar orifice, and was not far removed from the orifice of the duodenum, so that it was only the anterior portion of the intestinal canal that was dilated; the muscular fibres of the intestine could be easily traced, and were lost in the thick and vascular portion."

"On the outside of the sac, near the place where the funis entered, a great number of large vessels were observable; and on the inside of the sac, not far from the insertion of the funis, was a large arterial branch ruptured, from whence the hæmorrhage proceeded, which was the immediate cause of the lad's death." Page 23.

The internal structure of the fœtus was not examined. Such an omission is indeed somewhat unfortunate, for in this way alone could it have been rendered really subservivient to the interest of science. By peculiarity of structure or distribution of the internal organs, developed on such an examination, some obscure or questionable point in physiology might have been illustrated. We wish that our author had possessed somewhat of the philosophic spirit of Mr. Young. That gentleman did not trot up and down the country to exhibit his little monster, and forego, for the empty "honour of submitting it" to princes, the light of an interesting investigation. He did not publish his discovery in a splendid half-guinea quarto, with a long list of subscribers, and groupes of italics, and exclamations of wonderment and amaze!* No. His narrative, with that un-ostentatious calmness which distinguishes real science, was consigned in a publication where it will be read by those only who are capable of appreciating its value, and read long after the heart of Mr. Highmore shall have become insensible to the smile of royalty,— his ear dead to the fleeting hum of popular applause; and when nothing but the title-page of his pamphlet shall be left by time's desolating hand as a memorial that it once existed.

Far, however, be it from us to quarrel with Mr. Highmore for his lack of philosophy. It is not every man's lot to be made of philosophic stuff. We have accusations of a more serious and formidable nature to bring against him. Where was his discretion in not foreseeing the horrible consequences likely to result to certain of his Majesty's liege subjects from the exhibition of the headless monster in an isolated and popular form? Where his bowels of compassion towards the croakers of the human and ranine races? We have already heard of one poor gentleman, who, since the publication of Mr. Highmore's book, has discovered that his forty-years' fever and bowel complaint may be attributed to breeding; and keeps himself and all around him in a constant state of terror and alarm, with gloomy predictions and preparations for speedy accouche-

* There is something too much like trick in the mechanism of the book. Ten out of the thirty pages are occupied by the title-page, dedication, and list of subscribers.

ment. Nor is even criticism, in its coat of brass, proof against this terrible infection. For, on coming to page 14, one of our brethren threw down "the infernal book;" and with a face four inches and four degrees more rueful than common, begged of us *to examine his abdomen.* We have this moment carried him to his aerial abode, speechless and horror-stricken. Experiments also to elucidate the process of extra-uterine gestation are rapidly reviving among the beardless philosophers of Sherbone. Woe then to the flatulent and dyspeptic into whose hands this pernicious pamphlet may fall! Woe to the frogs of Dorsetshire!

Two engravings exhibit an anterior and posterior view of the fœtus and containing cyst. The latter is by far the most clear and expressive.*

After all, we are so stupid as to discover only one of the "several circumstances" in which, according to Mr. Highmore, his fœtus "materially" differs from the two whose organization and attendant phænomena we have rapidly traced in the commencement of this article. That circumstance is the motion felt in the tumor by the lad, and attested by his mother. But on evidence like this, no firm reliance can, perhaps, be placed. Mr. Highmore knows, or ought to know, the extreme circumspection with which the testimony of an invalid respecting his internal sensations should be received; nor are the asseverations of an ignorant spectator on a subject deeply interesting to the feelings, entitled to greater confidence: especially if that subject at all savour of the marvellous. Yet the circumstance is by no means improbable; and had the fœtus, on examination, dispayed a spinal marrow and nerves of voluntary motion, our scepticism on this score would have been well-nigh done away. In other respects, the Sherborne monster is decidedly less perfect than those of Paris and London: inferior to the former with respect to an encephalon; to the latter, in the number and configuration of its extremities.

Towards the conclusion of the work, a question is started by Mr. Highmore, whether or not the fœtus were originally deposited *in* the intestinal canal? Taking for granted the accuracy of his statements respecting the case, we

---

* We are surprised to read, in the explanations accompanying these engravings, the words *ilii* and *ischii*. In our school-days, nouns of the neuter gender invariably made *a* in the nominative plural. Such errors are indeed trifling; but they greatly disfigure the writings of *scientific* men.

feel little hesitation in answering in the negative. The ovum, consisting only of that vascular and spongy membrane, which subsequently formed the larger portion of the cyst, appears to have been at first deposited between the spine and posterior surface of the duodenum. As the tumor grew, it took, from the resistance of the vertebral column, an *anterior* direction; and encroached more and more on the intestine. At length, by continued pressure, a communication was formed between the cavities of the cyst and alimentary tube; and the fluid of the former and part of the fœtus passed through. Thus, the whole formed one sac consisting of two distinct portions. It seems probable, that this communication with the intestine took place subsequently to the 9th of May: for, on that day, "fluctuation" in the tumor was "very perceptible" The contained fluid was, probably, evacuated by the bowels and kidnies; as none was found on dissection in the cyst; and, about the middle of May, "an evident diminution in the circumference of the tumor was observed." This reasoning is confirmed by other considerations: Had the ovum been orginally deposited *in* the intestine, seven years would hardly have elapsed without some symptom, indicative of its presence on the highly organized and irritable mucous membrane: and, again, those laws by which the developement of internal tumors is governed, would have operated the increase of the sac in that direction where the least resistance existed; and the *anterior* not the *posterior*, wall of the intestinal tube would have yielded to its progress.

As to the speculations respecting the origin of the fœtus from "an unnatural crime," they seem worse than idle. If the professional friends of Mr. Highmore will have the goodness to recollect the very early period at which the abdominal tumor was noted in Bissieu and in Hare; if they be not prepared absolutely to disprove the inferences to which we have been directed by the light of physiology, that the female organs not only afford a nidus to the germ of the future progeny, *but contribute essentially to its formation;* we surely may hope to hear no more of these absurd fantasies and explanations, erroneously styled theories, by Mr. Highmore. The fact appears, as far as we are capable of reasoning upon it, to be simply this: Two ova, impregnated in the same female, become united by the operation of causes which we never shall nor can comprehend. Two fœtuses partially connected by the surface; the monster with two heads or supernumerary extremities; and the child, containing within its entrails the rudiments of a twin fœtus, probably constitute but various grades of this curious complication. Nothing more is proved by it than the phænomena of extra-uterine pregnancy have long since taught: an impregnated ovum will adhere and derive sustenance from any living structure with which it may be placed in contact. The impregnation and developement of the animal ovum, under ordinary circumstances, present a field of contemplation less curious, only because more common; and, in truth, quite as inexplicable. The nutrition of the little shrub which vegetates in our retirement, the structure of the feather with which we write, offer to the philosophic eye views and characters equally incomprehensible. For ever veiled from mortal ken are the mysteries of generation! No deviation from what we consider its ordinary laws will explain the secrets of the generative principle. The comet, by the infrequency of its appearance, excites the awe and admiration of nations; but it is subject to the same laws which regulate the march of our sober planet: and the philosopher, as he traces the wanderings of the aerial phænomenon, acknowledges its inadequacy to illustrate the essence of gravitation, and his own utter impotence to comprehend the causes by which the eccentricity of its path in the heavens was originally determined.

Yet cannot we take leave of Mr. Highmore without expressing our respect for the zeal and ability which he, on this occasion, has displayed. May they, ere long, be employed upon a more important topic! Our animadversions on his book may, at first sight, appear coarse and ungentle: we trust that he will find them, like strong physic, salutary. They have, at least, the grace of good intention. It is only against confirmed intellectual paralysis that we direct, in earnest, the titilations of the critical feather; only against the chronic maladies of the *heart*, that we shall use the blistering-fly and caustic. Such men as Mr. Highmore have little to fear from us. We honor his enthusiasm and his principles. But when time shall have tempered that enthusiasm, and allayed the effervescence of his senses, he will discern and acknowledge the justice of our observations: he will see the impolicy of decorating with ephemeral consequence a fact, which, however curious, is neither new nor susceptible of application to useful purposes; and lament, with us, that by a thirst of vulgar applause, he should have been allured from the sober and dignified path which science prescribed to his predecessors, and thus have brought his motives, pure and honorable as we believe they were, into suspicion or contempt with those, whose approbation is alone to be coveted,—whose censure is alone terrible.

