# Corrigenda

**Published:** 1816-02

**Authors:** 


					'' > '? J'0,Jr inches and, read four inches longer, an4

				

